# Application of Platelet-Rich Fibrin and Bone Morphogenetic Protein for Full-Mouth Implant-Based Oral Rehabilitation in a Case of Mandibular Osteoradionecrosis

**DOI:** 10.1155/2023/2449298

**Published:** 2023-05-30

**Authors:** Amirhossein Moaddabi, Parisa Soltani, Arman Yazdani, Mohammad Hossein Nikbakht, Pardis Amani Beni, Elahe Modabber, Flavia Iaculli, Gianrico Spagnuolo

**Affiliations:** ^1^Department of Oral and Maxillofacial Surgery, Dental Research Center, Mazandaran University of Medical Sciences, Sari, Iran; ^2^Faculty of Dentistry, Mazandaran University of Medical Sciences, Sari, Iran; ^3^Department of Oral and Maxillofacial Radiology, Dental Implants Research Center, Dental Research Institute, School of Dentistry, Isfahan University of Medical Sciences, Isfahan, Iran; ^4^Department of Neurosciences, Reproductive and Odontostomatological Sciences, University of Naples “Federico II”, Naples, Italy; ^5^Student Research Committee, School of Dentistry, Isfahan University of Medical Sciences, Isfahan, Iran

## Abstract

Osteoradionecrosis (ORN) is a debilitating complication following radiation therapy, which in the head and neck region, occurs most frequently in the mandible. Although ORN is rare, it is complex and multifactorial and requires appropriate management. Manipulation of bone in patients with head and neck cancers before radiotherapy can cause ORN. In this report, we aim to present successful insertion of four dental implants in the interforaminal segment combined with application of platelet-rich fibrin and bone morphogenetic protein in a 60-year-old male with stable ORN in the posterior regions of the mandible.

## 1. Introduction

Osteoradionecrosis (ORN) is a serious late consequence of radiation therapy, which affects the mandible more frequently than any other bone in the head and neck region [1]. The exact definition of ORN is a subject of debate. While several authors suggest that evidence of bone necrosis within the radiation field must be considered as ORN [2], and others argue that presence of persistent denuded bone for 3 months is a criterion for diagnosis of ORN. ORN occurs as a result of suppression of bone turnover due to a malfunction of osteoclasts, tissue hypoxia, and hypovascularity [2]. In addition, trauma to the affected bone area is an important factor for initiation of bone necrosis in the region [1].

Treatment of ORN is dependent on the severity of bone involvement [2]. The treatment comprises conservative approaches, including observation, drug therapy, debridement, and irrigation in milder cases and surgical resection combined with adjunct therapy in more severe cases [3, 4]. However, in general, the appropriate treatment modality for ORN is controversial. In addition, whether or not surgical rehabilitation in the affected bone can be successful, or counterproductive is largely related to the bone status and surgical protocols [1]. Different methods including application of platelet-rich concentrates and preoperative administration of hyperbaric oxygen and antibiotics have been proposed for prevention of ORN incidence after bone manipulation in the irradiated jaws [5, 6]. However, based on a Cochrane systematic review, due to the inadequate sample size and errors in reporting, the evidence on the potential of these agents for prevention from ORN is not certain [7].

Many patients who undergo radiotherapy lack healthy teeth and are unable to use conventional prostheses due to reduced saliva production as a result of radiation injury to the salivary glands [8]. For oral rehabilitation in these patients, prostheses that are supported by osseointegrated dental implants can be a suitable treatment option. However, ORN is considered a contraindication for dental implant placement. Lack of suitable bone in terms of height and width is the most common limitation of implant treatments. Autogenous block grafts, guided bone regeneration, use of growth factors, and tissue engineering are proposed to overcome these limitations [9, 10]. Additionally, to improve osseointegration, bioactive materials, such as platelet-rich plasma and platelet-rich fibrin (PRF), are available [11]. PRF is obtained from plasma after centrifugation of the blood. Leukocytes, platelet cytokines, and the fibrin matrix, which supports these elements are the most important reasons for its therapeutic effects [12]. Studies suggest that combining implant and PRF can lead to an acceptable regeneration of bone and might enhance implant stability during osseointegration [13]. Moreover, bone morphogenetic protein (BMP) is a growth factor that accelerates bone regeneration and was delivered as a bone growth additive in dental implant placement [14].

In this report, we aim to present successful insertion of four dental implants in the interforaminal segment combined with application of PRF and BMP in a 60-year-old male with stable ORN in the posterior regions of the mandible.

### 1.1. Case Presentation

The patient was a 60-year-old Iranian male seeking non-traumatic extraction of the remaining teeth, and regenerative treatments who was referred to an oral and maxillofacial surgeon (A.M.) in Sari, Iran. The patient had a previous history of advanced stage 4 squamous cell carcinoma of the right submandibular gland with unilateral mandibular involvement and metastasis to the lung that had been diagnosed at Amir Alam Hospital in Tehran in November 2018. Although the initial prognosis of the patient was poor, chemotherapy with cisplatin and docetaxel combined with radiation therapy to the submandibular region (70 Gy fractionated to 35 sessions of 200 cGy) had been completely successful in remission of both the submandibular tumor and lung metastasis in 45 days and, therefore, there was no need for surgical intervention. After that, the patient has been followed from 2015 to 2021 without any significant changes or recurrent disease. The patient had occasional pain episodes as a result of cancer therapy for which methadone was prescribed. The patient was not a smoker and did not consume alcohol.

**Figure 1:**
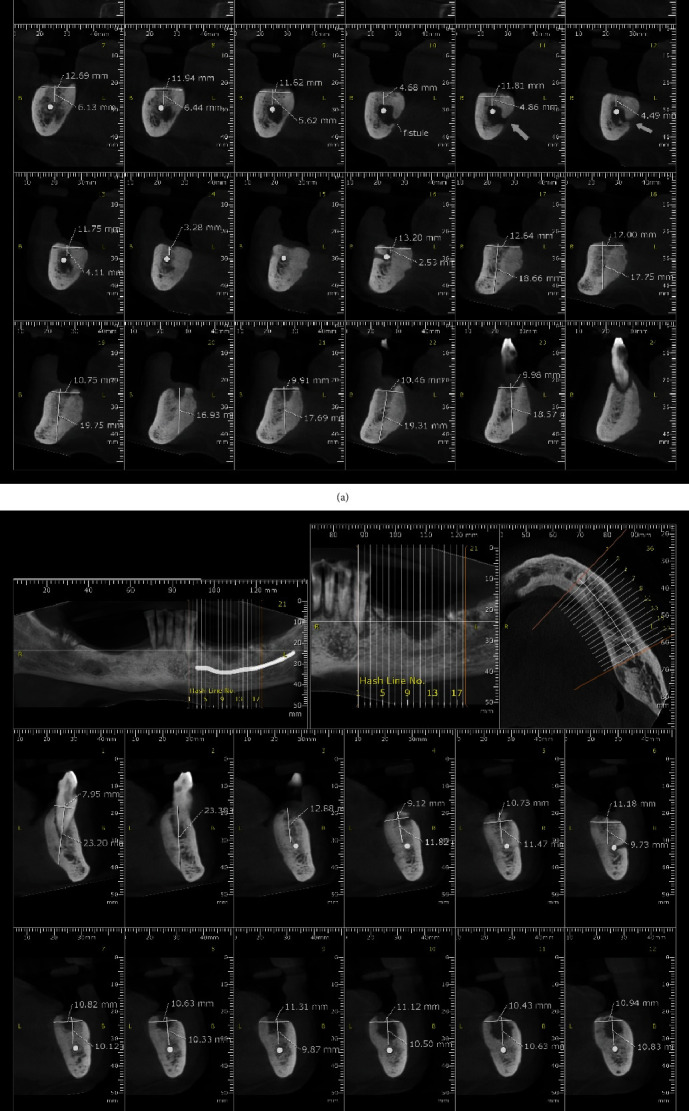
Pre-operative CBCT images of the patient depicting ill-defined patchy lytic radiolucent areas in the mandible surrounded by sclerotic bone in the posterior region on the (a) right and (b) left sides.

The patient complained of several symptoms following radiation therapy including burning sensation in the oral cavity, xerostomia, and gingival recession. In the extra-oral examination, the range of mouth opening and jaw movements was normal. In the intra-oral examination, the quality and quantity of saliva were normal. Oral soft tissue was normal without any areas of dehiscence, redness, or ulcer, and there were no signs of infection, pain, or oral fistulae probing to the bone. Additionally, no signs of exposed necrotic bone or mucosal inflammation was observed. Periodontal bone loss and caries with a pattern compatible with radiation caries were observed in the remaining teeth [15]. Panoramic radiograph and cone beam computed tomography (CBCT) scans of both jaws had already been obtained before the referral to the oral and maxillofacial surgeon. The radiographic images revealed ill-defined patchy areas of bone destruction in the mandible surrounded by sclerotic bone with a wide transitional zone on both right and left side in the posterior region. Sequesters were seen in the lesion, in addition to cortical bone resorption and a fistula to the right lingual cortical border (Figure 1). Differential diagnosis included ORN, chronic osteomyelitis, and diffuse sclerosing osteomyelitis.

**Figure 2:**
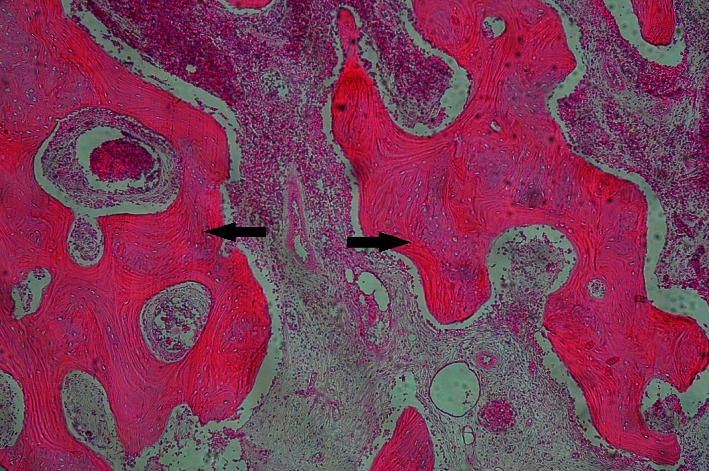
Hematoxylin and eosin staining of the incised sample indicating areas of bone necrosis (arrows).

An incisional biopsy was performed from the distal lesion on the right mandibular body using a small incision on the buccal surface close to the alveolar crest and curettage of the tissue. Histopathologic examination was performed using hematoxylin–eosin staining, and areas of bone necrosis were observed. Based on the radiological and histopathological findings as well as the history of radiation therapy of the mandibular region, a diagnosis of ORN was made (Figure 2). Since the ORN lesions were stable and without any clinical presentation, no treatment was planned for the lesions except for observation and follow-up.

Clinical examination revealed five anterior mandibular teeth with advanced periodontitis and several posterior mandibular roots as well as four anterior maxillary roots. Two loaded implants were present in the location of maxillary central incisors. The implants were inserted a few years ago and were asymptomatic and functional.

The patient asked for implant-based full-mouth prostheses in both jaws. The risks of failure and exacerbation of the condition as a result of surgical trauma were thoroughly discussed with the patient. Additionally, the patient was motivated to maintain good oral hygiene in the future. In order to evaluate the quality of the healing process, the mandibular teeth were extracted atraumatically without incision, using a flexible periotome and dental forceps with appropriate size. The 3-month follow-up was uneventful and revealed favorable healing. Therefore, after multiple sessions of discussion with the patient, implant-based overdenture of both jaws was planned. Due to the bone condition in the mandible and to reduce the amount of surgery, an overdenture based on four parallel implants in the interforaminal region was planned. For the maxilla, four new implants in the region of the canine and second premolar teeth in addition to the existing implants were considered. In order to enhance the chance of success, application of BMP, leukocyte PRF (L-PRF), and injectable PRF (I-PRF) was planned for the mandibular implants. Placement of the mandibular and maxillary implants was planned on two separate surgical sessions.

L-PRF and I-PRF were prepared in two stages prior to commencement of the surgery by obtaining 30 mL of blood from the patient. An hour before the surgical session, the patient took four amoxicillin 500 mg capsules and a tablet of ibuprofen + lysine 400 mg [16–19]. Then, immediately before the surgery, the patient rinsed his mouth with chlorhexidine 0.2% mouthwash [16].

After local anesthesia, a full-thickness flap was elevated in the mandibular alveolar ridge in the interforaminal region of the mandible with one small vertical release. In order to preserve maximum vascularity of the bone, elevation of the flap was performed using a minimally invasive approach. The drilling was then performed (1000 rpm, 35 Nm), and the parallel implants were placed. During the drilling, minimal bone bleeding was observed, and the bone surrounding the implant was fine and sclerotic. Two-stage implant insertion was planned for the patient. Bone-level implants (UF, DIO, South Korea) were prepared for insertion.

**Figure 3:**
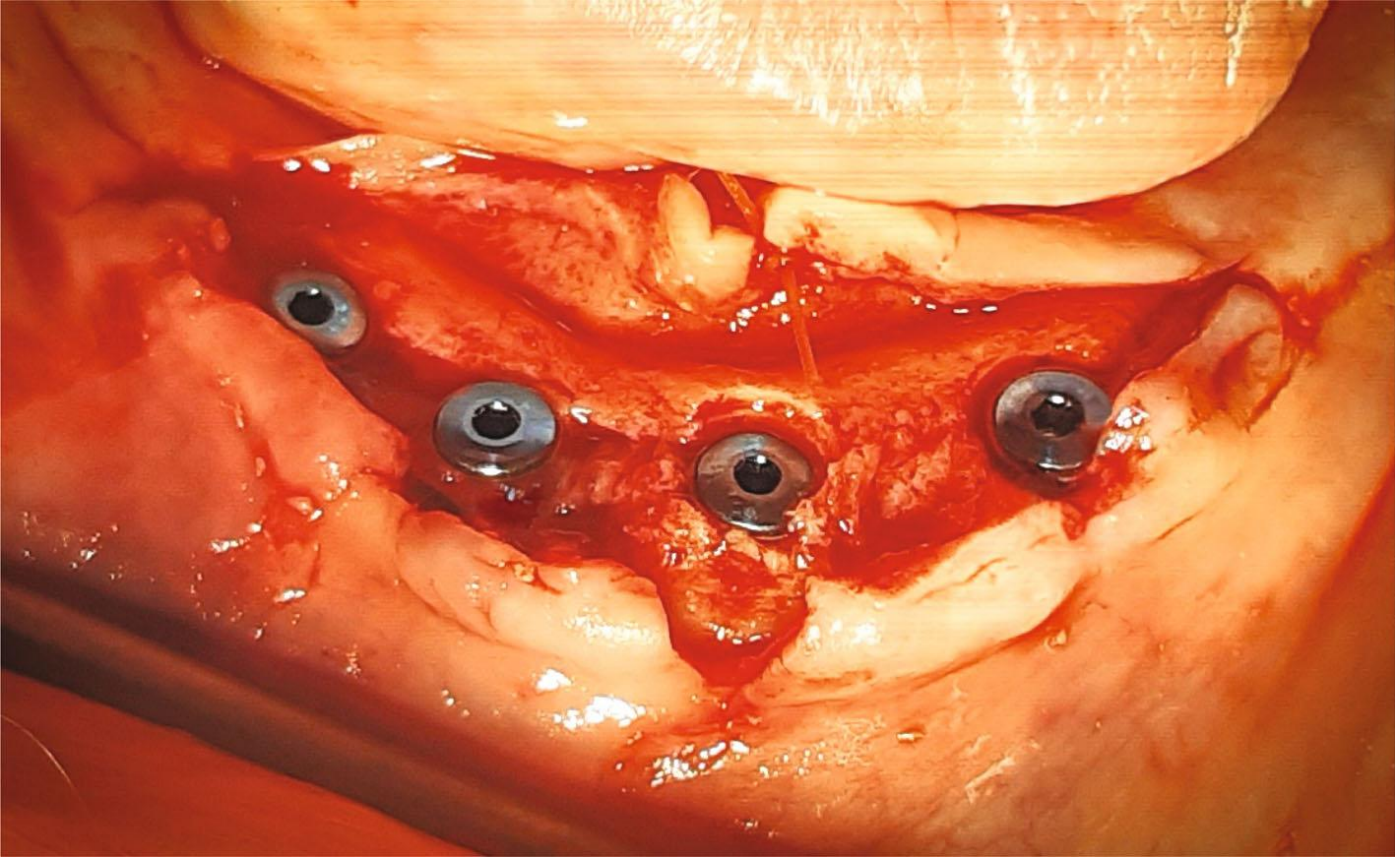
Post-surgical photograph of the inserted mandibular fixtures in the anterior region.

In order to produce L-PRF, 10 mL of blood was obtained from the patient and immediately poured in a glass tube without any anticoagulant agent. The blood was centrifuged (IntraSpin, Biohorizons, USA). For I-PRF the same procedure was followed, but the blood was poured in the special plastic tube. In addition, three 0.1 g pieces of recombinant human BMP (RHBMP2, Cowellmedi, South Korea) were mixed with sterile distilled water according to the manufacturer's instructions. Four implants (two with 4 mm × 13 mm and two with 4 mm × 10 mm dimensions) were then inserted (35 rpm, 35 Nm), and the cover screws were fastened after checking the primary stability (Figure 3).

**Figure 4:**
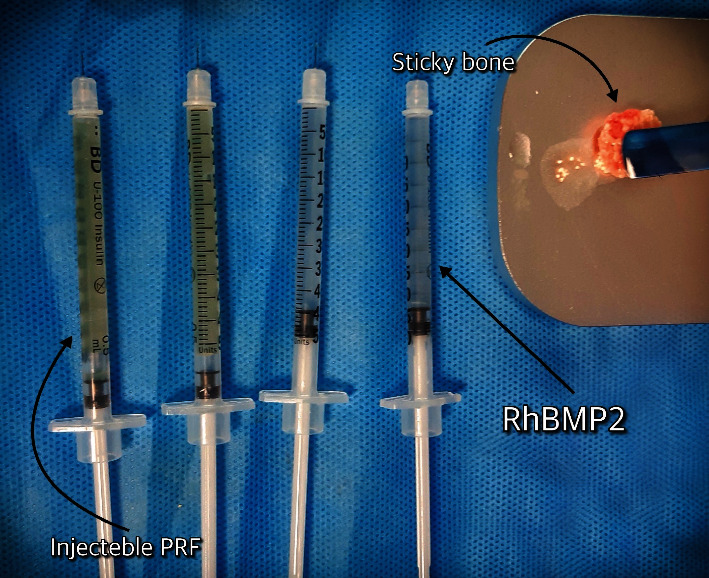
Prepared injectable PRF, recombinant human BMP 2 (RhBMP2), and sticky bone (combination of leukocyte PRF, xenograft, and autogenous bone particles obtained by drilling).

The surgical site was thoroughly rinsed with normal saline. Sticky bone was then prepared by combining L-PRF, xenograft (BoneB+, Iran), and autogenous bone particles obtained by drilling. The resultant sticky bone was placed in labial and crestal regions and covered with an autologous membrane obtained from L-PRF. The incision was closed using Vicryl rapid 4-0 suture (Ethicon, USA) without tension. RHBMP2 and I-PRF were injected from the buccal side into the wound area (Figure 4).

Amoxicillin 500 mg and metronidazole 250 mg every eight hours was administered for 1 week [16]. Ibuprofen + lysine 400 mg was administered every six hours for 1 week [20]. Chlorhexidine 0.2% mouthwash was also recommended three times a day for 1 week [21].

**Figure 5:**
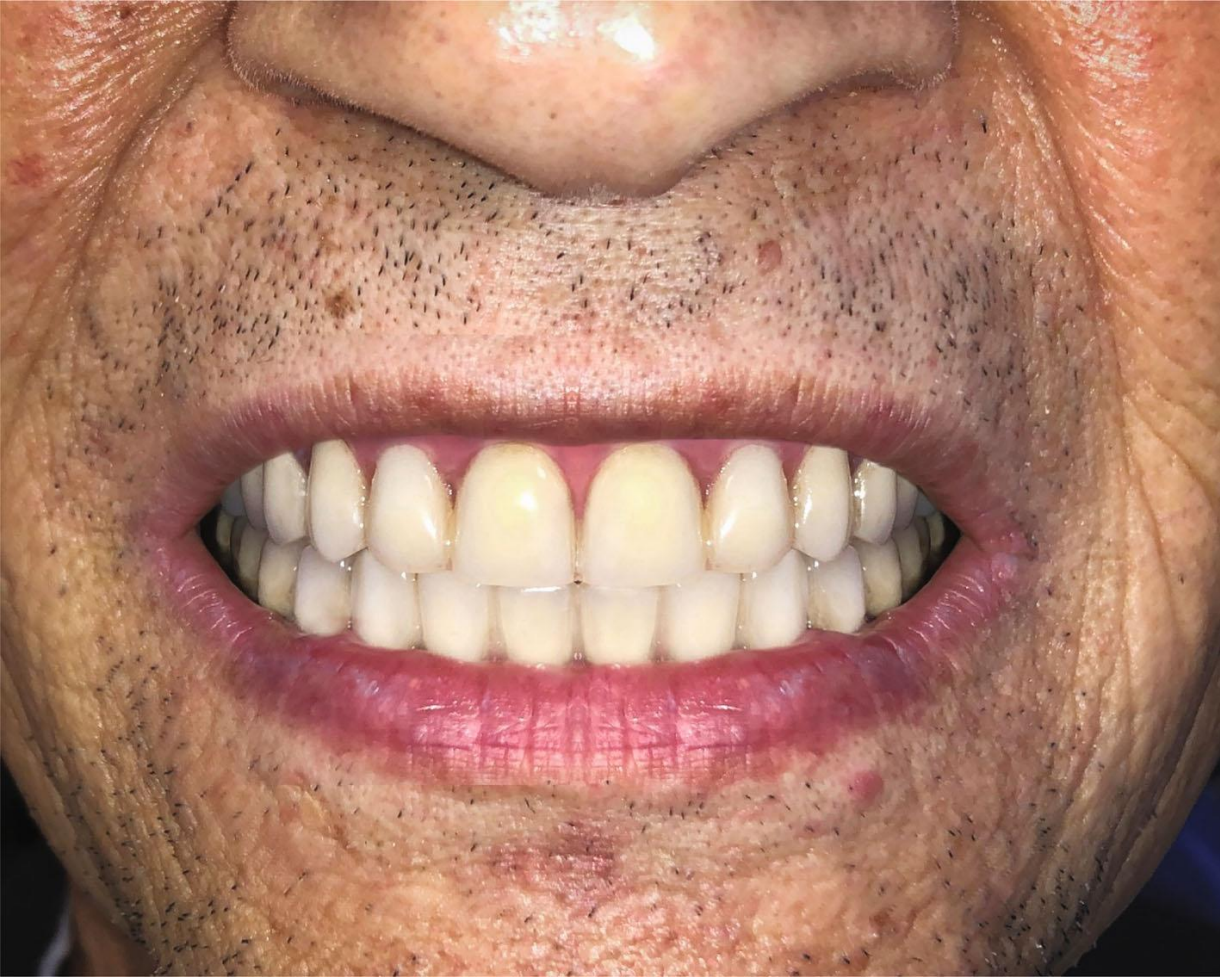
Intra-oral photograph depicting the final results of full-mouth reconstruction.

The next day, the patient was contacted. He reported an uneventful recovery and no pain or other symptoms. Seven days after the surgery, the surgical site was examined. No evidence of dehiscence or infection was observed, and the oral hygiene was appropriate. In the fourth-month of follow-up, exposed cover screws were observed, and the patient was asked to regularly cleanse the area with normal saline. The maxillary implants (two with 3.8 mm × 13 mm and two with 4.5 mm × 10 mm dimensions) were placed in this session following the same protocol except for application of RHBMP2. Three months after placement of the maxillary implants, a panoramic radiograph was obtained revealing successful osseointegration of all implants in both jaws. The patient was then referred to the prosthodontist for construction of the full-mouth prostheses using bar attachments and overdentures for both jaws (Figure 5).

**Figure 6:**
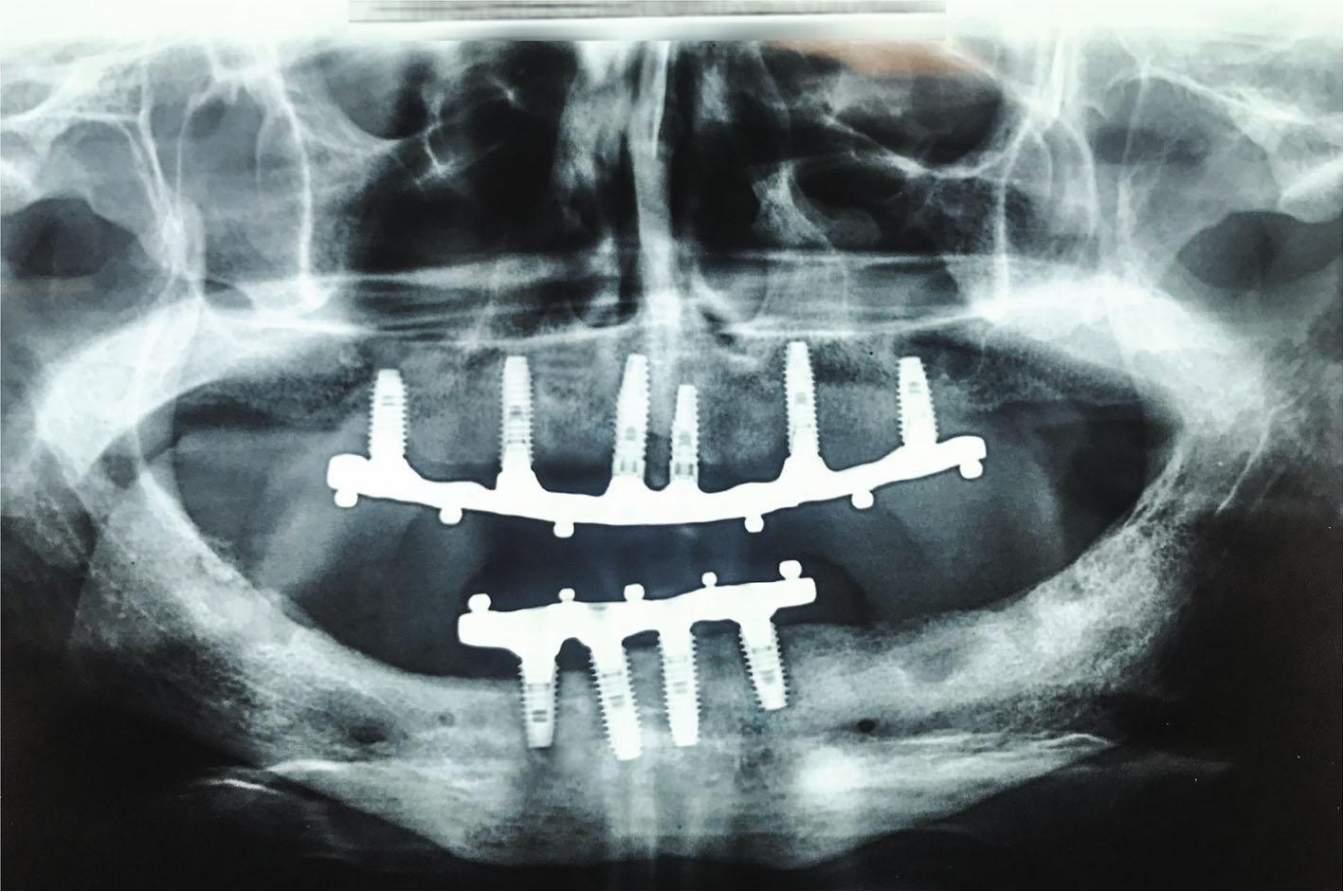
Follow-up panoramic radiograph 10 months after loading.

**Figure 7:**
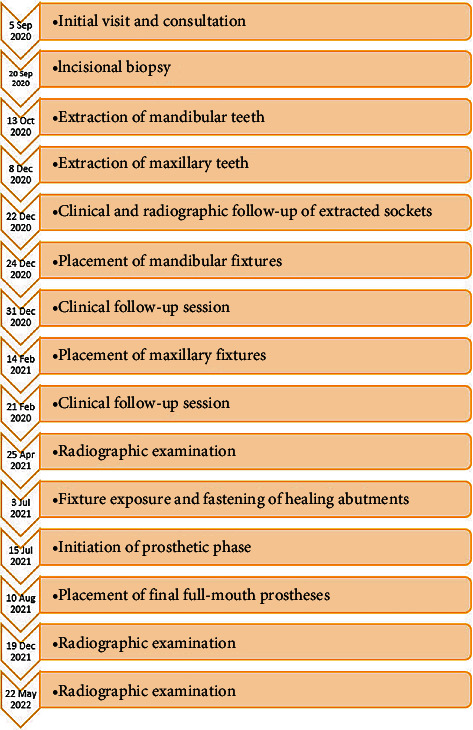
Timeline of the case.

The patient was followed-up again 5 and 10 months after loading of maxillary and mandibular implants by clinical and panoramic radiograph examinations. In addition to favorable clinical and self-reported findings, no evidence of implant failure, considerable peri-implant bone loss, or deterioration of the osteonecrosis regions was observed in any of the radiographic examinations (Figure 6).  Figure 7 illustrates a timeline of the case.

## 2. Discussion

ORN is currently considered as a contraindication for placement of dental implants. Nevertheless, the present case opted for implant-based treatments. However, it was only after observing the favorable healing of the extracted mandibular sockets, that insertion of implants in the anterior segment of the mandible was planned. The mandibular anterior region did not show any evidence of bone necrosis and, therefore, insertion of four parallel implants was considered in the interforaminal region. However, in order to improve the bone regeneration, application of a sticky bone compound made from autologous bone particles, xenograft material, L-PRF, and covered by autologous membrane as well as injection of I-PRF and RHBMP2 was performed. The patient was completely aware of the risks of implant-based treatments due to his condition. Although at first a removable acrylic denture was offered as the treatment of choice, the patient chose implant-based overdenture prosthesis considering its benefits. Additionally, he was motivated to adhere to oral hygiene procedures and regular follow-up sessions.

PRF is composed of a polymerized fibrin matrix with incorporated platelets, leukocytes, and cytokines, and circulating stem cells that can enhance healing of soft and hard tissues [22]. The findings of Strauss et al. show that PRF creates a therapeutic potential for wound healing and regeneration by cell proliferation, adhesion, differentiation, and inflammation [23]. PRF causes an increase in osteoblastic attachment, enhanced proliferation by the Akt pathway, and synthesis of the matrix through activation of heat shock proteins and collagen-synthesis proteins. This can ultimately lead to enhanced bone healing and regeneration [24]. Chen and Chang in their case report presented favorable treatment outcomes using a combination of PRF application and sequestrectomy for treatment of mandibular ORN [25]. In a recent literature review, Harris et al. reported that application of PRF as an adjunct to surgical procedures is beneficial for treatment of ORN lesions [26]. In the present report, although the implants were placed in an area anterior to the bone necrosis region, PRF was used as a regenerative agent to improve bone healing and response. During the healing process, the ability of PRF to gradually release growth factors into the surrounding bone was utilized. Additionally, RHBMP2, which has shown to improve bone regeneration was used for the present case [14]. Although some reports have shown that using PRF as the sole filling material, generated new bone in the affected areas [27, 28]. Additionally, application of growth factors may improve the post-operative outcome of surgical treatment of drug-related osteonecrosis of the jaws [29].

According to the literature, using PRF can increase the quality of the newly formed bone and enhance the bone formation rate (Ozdemir et al., 2013). Another study concluded that the combination of the xenogenic bone substitute mineral with BMP can enhance the maturation process of bone regeneration and can increase the graft to bone contact in humans [14]. The final results of this case report were in line with these findings in the literature.

The last follow-up of the patient was 10 months after loading of maxillary and mandibular implants. All the clinical, self-reported, and radiographic findings in all of the follow-up sessions were indicative of appropriate healing and regeneration of implant sites, no significant marginal bone loss in any of the inserted implants, functionality of dental implants and overdentures, and stable condition of the areas of osteonecrosis in the posterior segments of the mandible. The patient will be followed up annually afterward. As a case report, the clinical outcome observed in the present patient cannot be generalized to other instances. However, the present case provided valuable initial evidence for the successful application of bioactive material in patients with compromised bone status. Furthermore, randomized controlled trials are needed to demonstrate the efficiency of these adjunct treatments in alveolar bone affected by ORN. Additionally, the relatively short follow-up duration was a limitation for this case report.

## Data Availability

Due to the patient's confidentiality, only anonymous data can be shared upon reasonable request from the corresponding authors.
